# Exploratory use of cardiac magnetic resonance imaging in liver transplantation; a one-stop shop for preoperative cardio-hepatic evaluation

**DOI:** 10.1186/1532-429X-13-S1-P137

**Published:** 2011-02-02

**Authors:** Asghar A Fakhri, Ngoc L Thai, Jose Oliva, Michael K Dishart, Bryan Veynovich, June A Yamrozik, Ronald B Williams, Saundra B Grant, Jacqueline Poydence, Vikas K Rathi, Anil Singh, Kusum B Tom, Swami Nathan, Robert WW Biederman

**Affiliations:** 1 Allegheny General Hospital, Pittsburgh, PA, USA; 2 Bon Secours Heart and Vascular Institute, Richmond, VA, USA

## Introduction

Preoperative cardiovascular (CV) risk stratification in liver transplant (LvTx) candidates has proven challenging due to limitations of current noninvasive modalities. Additionally, the evaluation can be cumbersome and expensive given the need for separate cardiac, vascular and abdominal imaging. Over the last decade cardiovascular MRI (CMR) has emerged as the 'gold standard' for many important CV metrics used to define such risk due to its unparalleled spatial resolution, lack of ionizing radiation and intrinsic 3D capabilities.

## Purpose

Hypothesis We hypothesize that it is feasible to perform a CMR driven preoperative cardiohepatic evaluation in LvTx candidates as a ‘one-stop shop’ in a dedicated CMR suite.

## Methods

In this pilot study, patients (pts) underwent left/right ventricular function assessment (SSFP), stress CMR, and thoracoabdominal MRI/MR angiography (MRA). Pharmacologic stress CMR was done with regadenoson, adenosine, or dobutamine. Viability was assessed by delayed hyperenhancement (DHE). Cardiologists managed pts during acquisition and interpreted CMR studies. Diagnostic radiologists interpreted abdominal MRI/A.

## Results

Over 2 years, 51 LvTx candidates (mean age 56 years, 35% female; mean MELD score of 14, Child-Pugh Class ≥ B) underwent CMR with an average imaging time of 72±23 minutes. This included 7 pts on mechanical ventilation and 6 on vasopressors for shock. All pts completed SSFP CMR, 98% completed stress CMR, 82% completed DHE viability (3 renal failure pts underwent dobutamine CMR), 94% completed liver MRI, and 88% completed MRA. Four pts had coronary angiography (3 for ischemia on CMR and 1 for post-operative ischemia), and none had flow-limiting coronary disease. Nine pts underwent orthotopic LvTx (mean 74 days to LvTx after MRI). There were 7 ascertained deaths in the non-LvTx group (mean 23 days post CMR) and 1 death in the LvTx group (116 days after MRI, 11 days after LvTx). Average cost saving was >$1500/per pt and >2 days of additional testing.

## Conclusion

It is feasible and efficient to perform comprehensive preoperative liver transplant imaging in a CMR suite, even in critically ill patients. Future evaluations will systematically focus on prognostic accuracy, patient convenience, and cost effectiveness.

